# Suubi4Stigma study protocol: a pilot cluster randomized controlled trial to address HIV-associated stigma among adolescents living with HIV in Uganda

**DOI:** 10.1186/s40814-022-01055-7

**Published:** 2022-04-29

**Authors:** Proscovia Nabunya, Fred M. Ssewamala, Ozge Sensoy Bahar, Lynn T. M. Michalopoulos, James Mugisha, Torsten B. Neilands, Jean-Francois Trani, Mary M. McKay

**Affiliations:** 1 International Center for Child Health and Development (ICHAD), St. Louis, USA; 2 Brown School, Washington University in St. Louis, Campus Box 1196, One Brookings Drive, St. Louis, MO 63130 USA; 3 The Moving Well Project International, Inc., Silver Spring, USA; 4 School of Social Work, Columbia University, New York City, USA; 5 College of Health Sciences, Makerere University, Kampala, Uganda; 6 Division of Prevention Science, School of Medicine, University of California San Francisco, San Francisco, USA

**Keywords:** HIV-associated stigma, Children and adolescents living with HIV, Multiple family group, Group cognitive behavioral therapy, Uganda

## Abstract

**Background:**

Sub-Saharan Africa (SSA) is heavily burdened by HIV, with 85% of the global new infections among adolescents happening in the region. With advances in medication and national policies promoting antiretroviral therapy (ART), children < 15 years living with HIV (CLWH) continue to grow with a chronic, highly stigmatized disease. Unfortunately, the stigma they experience results in much lower quality of life, including poor mental health and treatment outcomes. Family members also experience stigma and shame by virtue of their association with an HIV-infected family member. Yet, stigma-reduction interventions targeting CLWH and their families are very limited. The goal of this study is to address HIV-associated stigma among CLWH and their caregivers in Uganda.

**Methods:**

This three-arm cluster randomized control trial, known as *Suubi4Stigma,* will evaluate the feasibility, acceptability, and preliminary impact of two evidence-based interventions: (1) group cognitive behavioral therapy (G-CBT) focused on cognitive restructuring and strengthening coping skills at the individual level and (2) a multiple family group (MFG) intervention that strengthens family relationships to address stigma among CLWH (*N* = 90, 10–14 years) and their families (dyads) in Uganda. Nine clinics will be randomized to one of three study arms (*n* = 3 clinics, 30 child-caregiver dyads each): (1) usual care; (2) G-CBT + usual care; and (3) MFG + usual care. Both treatment and control conditions  will be delivered over a 3-month period. Data will be collected at baseline (pre-intervention) and at 3 months and 6 months post-intervention initiation.

**Conclusion:**

The primary aim of the proposed project is to address the urgent need for theoretically and empirically informed interventions that seek to reduce HIV-associated stigma and its negative impact on adolescent health and psychosocial well-being. As several countries in SSA grapple with care and support for CLWH, this study will lay the foundation for a larger intervention study investigating how HIV-associated stigma can be reduced to foster healthy child development—especially for CLWH as they transition through adolescence.

**Trial registration:**

ClinicalTrials.gov: NCT04528732; Registered August 27, 2020

## Introduction

Globally, an estimated 1.8 million children < 15 years are living with HIV [1, 2]. Sub-Saharan Africa (SSA) is heavily burdened by HIV, with 85% of new infections among adolescents happening in the region [2]. Uganda is one of 7 countries in SSA to achieve the 90-90-90 testing, treatment, and viral suppression targets [2, 3]. However, even with these improvements, HIV prevalence is still high (7.5%) among people between 15 and 49 years [4]. Moreover, close to 150,000 children (ages 0–14) were living with HIV in 2019 [2]. While availability and access to free antiretroviral therapy (ART) have decreased child mortality [5], such accomplishment has resulted in the likelihood that more children living with HIV (CLWH) will transition into adulthood with HIV, a chronic, highly stigmatized illness [6]. Unfortunately, the stigma they experience results in a lower quality of life. However, very few stigma-reduction interventions targeting CLWH and their families exist in SSA [7, 8]. Thus, there is a need for data driven research to address stigma, especially among CLWH as they transition through adolescence into young adulthood.

### HIV-related stigma and associated outcomes

Among people living with HIV (PLWH), stigma is a common experience characterized by public blame and moral condemnation for contracting the infection [9–11]. It perpetuates a culture of silence and fear and prevents individuals from testing and seeking health care [9]. Stigma can be internalized as a result of perceived negative public attitudes. It translates into feelings that the self is reprehensible, damaged and defective; and is associated with depression and post-traumatic stress disorders (PTSD) [12, 13], feelings of loneliness and social isolation [14, 15], poor treatment and adherence to medication [10, 16], poor HIV-related physical health [17], and increased sexual risk-taking behavior [18]. Moreover, internalized stigma increases the risk of loss to treatment follow up [19]. Public stigma is manifested by the general population through negative stereotypes such as those related to sexual behaviors, prejudice (fear, aversion, hatred), and discrimination, all of which create social barriers, including access to healthcare [20]. Moreover, many CLWH live with extended family members after losing their parents to HIV, where stigma is perpetuated through rejection, verbal insults, physical abuse, avoidance and ostracism due to unfounded fears of infection [21].

At the family level, family members are often condemned and stigmatized in similar ways, by virtue of their association with an HIV infected family member [22, 23]. Stigma at the family level may be manifested through gossip, name calling, rejection and social isolation, loss of social support, and harassment [22, 23]. Specifically, family members are often held accountable for not preventing the perceived “immoral behaviors” of the HIV-infected family member, leading to feelings of failure, anger, guilt, and shame [22]. Such feelings negatively affect family caregiving roles, family functioning, and HIV health outcomes for PLWH, including CLWH. Due to this environment, CLWH may miss developing strong attachment bonds with family members and fail to develop self-esteem, emotional, and behavioral regulations [24]. Such unsupportive social environments increase the risk for mental disorders, including depression, stress, and anxiety [25]. Therefore, it is critical to develop HIV stigma reduction interventions to improve life satisfaction, family functioning, and reduce the potential spread of HIV.

### Potential role of family members in addressing HIV-associated stigma

Adolescence is a period of multiple vulnerabilities marked by the onset of physical and emotional maturation accompanied by the challenges of adapting to social, emotional, and cognitive changes [26]. Hence, children need additional support, including emotional support and acceptance from family and community members. Yet, many CLWH cannot count on the “normal” transition to adolescence due to HIV-associated stigma where community and family members ostracize them for being HIV positive [27], and where family members suffer the same treatment due to having a CLWH in the family [22, 23]. Thus, understanding the role of family members, and involving them in the design and implementation of family level HIV-related programs and interventions for CLWH is essential to their success.

The protective role of families in influencing children’s behavior and mental health outcomes has been well documented [28]. Studies have shown that quality of family relationships predict child mental health functioning and overall adjustment, and that when families are consistently involved in children’s lives, children experience a more positive transition through adolescence [29, 30]. Parental skills have been associated with children’s psychological adjustment, less risky sexual behavior, and less susceptibility to peer pressure [31, 32]. Moreover, more frequent and open communication with parents is associated with better psychological adjustment [33, 34]. Yet, for CLWH, parent-child communication and involvement may be adversely affected by orphanhood and HIV-associated stigma. Therefore, family support strategies may strengthen family functioning and help to address individual and family-level stigma.

### Need for HIV stigma reduction interventions targeting CLWH

While the negative impacts of HIV-associated stigma have been well-documented, systematic reviews have found that stigma reduction interventions for CLWH in SSA are almost non-existent [7, 8]. Existing interventions focus on reducing fear of HIV infection among non-infected populations [8] as they interact with PLWH. For example, of the 48 stigma reduction interventions, only 3 aimed to reduce stigma among PLWH in SSA [35, 36]. None targeted CLWH, nor assessed the impact of stigma reduction on HIV-related outcomes. To address these gaps, this pilot trial, entitled *“Suubi4Stigma”* (also known as *Hope for Stigma* in Luganda local language), seeks to address the urgent need for innovative, theoretically, and empirically informed interventions to reduce HIV-associated stigma and its negative impact on adolescent health and psychosocial well-being. This study examines two evidence-based interventions used in mental health settings, schools, and communities: (1) group cognitive behavior therapy (G-CBT) focuses on cognitive restructuring and strengthening coping skills at the individual level; and (2) a multiple family group (MFG) intervention that strengthens family relationships to address stigma among CLWH and their families. The specific aims are the following:

#### Aim 1

Pilot test the feasibility, acceptability, and preliminary impact of G-CBT and MFG on reducing HIV-associated (internalized and family level) stigma, and its impact on adolescent and family outcomes (trauma symptoms, depression, sexual risk, family/social support and adherence to medication) compared to: (a) usual care vs G-CBT; (b) usual care vs MFG; (c) G-CBT vs. MFG.

#### Aim 2

Qualitatively examine participants’ and facilitators’ intervention experiences and identify individual, family, and institutional-level facilitators and barriers to G-CBT and MFG intervention implementation and participation.

The Suubi4Stigma study has been designed as a three-arm cluster randomized control trial (RCT), consisting of a control arm and two treatment arms. Assessments will be conducted at baseline, 3 months, and 6 months post-intervention initiation.

### Theoretical framework

This study is informed by the HIV stigma framework [37, 38] suggesting that HIV stigma impacts PLWH via distinct HIV stigma mechanisms of internalized, anticipated, and enacted HIV stigma. Anticipated and enacted HIV stigma involve experiences with others [38]. Internalized stigma—the focus of this study—involves endorsing negative feelings and beliefs associated with HIV and applying them to the self. In addition, family members of PLWH are also subjected to and experience stigma by association via similar mechanisms. Within this framework, MFG provides opportunities for caregivers and children to communicate in a safe setting. It focuses on addressing internalized and family-level stigma by normalizing shared experiences with other families, fostering peer support and family communication, facilitating optimism and morale, and enhancing interpersonal and coping skills [39].

**Fig. 1:**
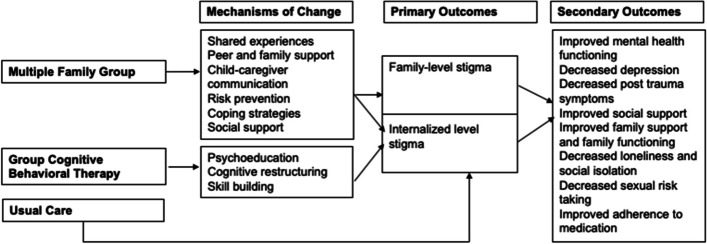
Conceptual model

On the other hand, G-CBT addresses internalized stigma through the core components of psychoeducation, cognitive restructuring, and skill-building to increase adaptive coping mechanisms [40]. These mechanisms may impact a range of psychological, behavioral, and health outcomes for CLWH and their families (Fig. 1).

## Methods

**Fig. 2:**
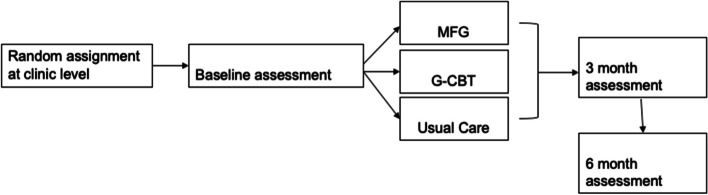
Suubi4Stigma study design

The Suubi4Stigma study is a three-arm cluster RCT evaluating the feasibility, acceptability, and preliminary impact of G-CBT versus MFG interventions among, 90 CLWH (10–14 years) and their caregivers (dyads). Nine clinics will be randomized to one of three study arms (*n* = 3 clinics, 30 child-caregiver dyads each): (1) usual care; (2) G-CBT + usual care; and (3) MFG + usual care. Both treatment and control arms will be delivered over a 3-month period. Data will be collected at baseline (pre-intervention), 3 months, and 6 months post-intervention initiation (Fig. 2).

### Study setting

The study will be implemented in the greater Masaka Region of Uganda. The region is composed of seven political districts: Rakai, Masaka, Lwengo, Kalungu, Lyantonde, Kyotera, and Bukomansimbi, and has the highest HIV prevalence compared to the national average [3, 4]. Child-caregiver dyads will be recruited from health clinics where the Washington University’s International Center for Child Health and Development (ICHAD) that houses the study and Reach the Youth (RTY), our implementation partner, operate. For a health clinic to be included in the study, it will have to be credited by the Uganda Ministry of Health to provide ART and have adolescent friendly services (e.g., adolescent days).

### Study participants

A total of 90 CLWH and their caregivers (dyads) enrolled in care at a health clinic that has partnered with ICHAD and RTY will be recruited into the study. Child inclusion criteria are: (1) living with HIV and know their status, (2) prescribed ART, (3) living within a family (defined broadly—not necessarily with biological parents), and (4) between 10 and 14 years. All eligible children from a particular household will be enrolled in the study and will be assigned to the same study condition. In addition, caregivers of children who agree to participate in the study will be enrolled. If the caregiver chooses to withdraw from the study, the child will also be asked to withdraw from the study. Children and caregivers will be excluded if they are unable to comprehend the informed consent/assent process and study expectations or unwilling to complete study activities. A complete timeline of project activities is presented in Table 1.

**Table 1:** Study timeline

Activities	1-3	4-6	7-9	10-12	13-15	16-18	19-21	21-24
Study preparations (hiring & training of research staff, refinement/adaptation of MFG and G-CBT interventions, preparation/refinement of assessment instruments, development of interview protocol, IRB approval)	X							
Participant recruitment and baseline assessments; intervention training for facilitators (MFG and G-CBT)		X						
Intervention delivery (MFG and G-CBT)			X					
3-month assessment (post-intervention), including qualitative interviews				X				
6-month assessment (follow-up)					X			
Quantitative data entry		X	X	X	X	X	X	
Qualitative data transcription and translation				X	X			
Data analysis, report writing, and results dissemination				X	X	X	X	X

### Clinic randomization

Stratified random sampling will be utilized to assign health care clinics to two strata based on health clinic level (health center III vs health center IV) to ensure overall clinic balance across study arms. Per the 2019 Uganda Ministry of Health’s Guidelines for Health Center Health Unit Management Committees (http://library.health.go.ug/publications/health-infrastructure), Health Center III is amid level care with provisions for basic laboratory services for diagnosis, maternity care and inpatient care with first level referral cover to the subcounty. It is usually staffed by nurse aids, qualified nurses and clinical officers. Health Center IV on the other hand, is a higher-level primary care facility below the district hospital, staffed with a medical doctor, with provisions for operating theater, inpatient and laboratory services and receives referrals from lower-level health facilities. All clinics offer HIV services. Each clinic will be randomly assigned to one of the three study arms, such that all selected child-caregiver dyads in the same clinic receive the same intervention, to reduce contamination. Randomization of clinics will be done by an independent research associate based at Washington University in St. Louis, using STATA software. Each health clinic’s assignment to a study condition will remain blinded to other study participants and facilitators, with the exception of research assistants, Project Coordinator and MPIs.

### Recruitment, retention, and attrition

Recruitment procedures tested in our previous studies will be utilized [41]. Children will be identified and recruited from health clinics associated with RTY and ICHAD. Patients are seen at least annually and each patient on ART must have prescriptions filled monthly at each of the health clinics. Although appointment days (not times) are provided, most patients arrive early in the morning on days that are convenient for them and wait for several hours before they are seen, providing an opportunity for recruitment through medical staff. A list of all eligible families will be created from medical records by one of the medical staff. Each chart contains data on the patient’s HIV status, age, and family. A clinic staff member will review the daily schedule, noting the eligibility of patients. Providers will present the project to adult caregivers of eligible children during appointments. If caregivers are interested, verbal consent to be contacted by research staff (who will be on-site during clinic days) will be requested. After speaking with the research staff one-on-one about the study, interested caregivers will provide written consent for themselves and for their child to participate. Children will be asked to provide written assent separately to avoid coercion.

The Suubi4Stigma study will take place in a highly stable region where geographical moves are rare, facilitating the ability to track and retain the sample. Retention procedures used in our previous Suubi studies will be utilized [41–43]. Specifically, we will ask participants to give their telephone number (if they have one), names, addresses, and contact information for three people who will always know how to reach them. Participants will be told that if we contact the people listed, we will not discuss any details about them or their study participation. We will use these records to help track their location only if we have lost contact. In addition, we will have monthly contact with children in both treatment conditions during MFG and G-CBT intervention delivery. This frequent contact will enable the research team to continually engage all participants and minimize loss to follow-up. Based on prior studies in the same region, we conservatively expect attrition by end of the 2-year study to be no more than 10% [43, 44].

### Suubi4Stigma study arms

#### Control arm (usual care)

All participants in both control and treatment arms will receive the traditional clinic intervention that focuses on testing services, as well as medical and treatment support for PLWH, including children and adolescents [45]. Currently, patients coming to the clinics receive testing and ART treatment as well as information about disease management. Both children and caregivers will receive this information. All participants in both control and treatment arms will receive medical and psychosocial support as part of usual care.

#### Treatment arm 1 (G-CBT)

In addition to usual care, participants in this arm will receive 10 sessions of G-CBT for HIV-associated stigma. Within G-CBT for stigma, we will utilize core components of CBT, such as psychoeducation, cognitive restructuring, and skill-building to increase adaptive coping mechanisms [40]: (1) exploration of HIV’s role and impact of stigma in adolescents’ lives; (2) use of cognitive restructuring to identify and address the negative stigma-associated beliefs, loss of self-esteem, and self-blame; and (3) skills-building around stress management and emotion-focused coping strategies to address negative feelings (e.g., assertiveness, relaxation skills and problem solving skills) [46]. G-CBT will be facilitated by trained para-counselors with experience in mental health support and working with children and adolescents living with HIV in the study region. Caregivers will not participate in G-CBT. Sessions will be delivered twice a week, outside of school hours and will be delivered in the Luganda local language. G-CBT is likely to offer more opportunities for normalization, positive peer modeling, reinforcements, social support, and exposure to social situations and feedback sources, given the context of shared experiences [47].

#### Treatment arm 2 (MFG)

The MFG intervention is rooted in family systems theory, structural family theory and social learning theory with elements of psychoeducation and social group work. MFG is a family-centered, group-delivered, evidence-informed, strength-based 10-session intervention for adolescents whose families struggle with poverty and associated stressors [39]. MFG integrates components of existing evidence-based practices that successfully improve parental management, mental health promoting family processes, and family strengthening [48, 49]. Specific session content will draw on the current interventions implemented by ICHAD [42, 50]. Sessions will focus on the core components of MFG, also known as 4Rs and 2S’s (rules, responsibility, relationships, respectful communication, stress, and social support). Families (children and caregivers) will be combined into groups of no more than 10 families each to promote communication and support within and among families. Sessions will be delivered in the Luganda local language, will last approximately 1 h and will be delivered twice a week, outside of school hours. Given the significant and protective role families play in children and adolescents’ health and mental health, we expect that strengthening family functioning and dialogue by involving caregivers through MFG will lead to better child outcomes, including addressing HIV-associated stigma.

### Intervention adaptation, delivery, monitoring, and fidelity

We will engage stakeholders, including implementing partners, community health care workers and peer parents already trained in the delivery on MFG sessions [42, 50], as well as in-country mental health workers and community leaders, and para-counselors, who will deliver G-CBT sessions. In Uganda, para-counselors are trained to assist with the psychological needs of individuals, including those related to HIV/AIDS and mental health [51, 52]. Specifically, while counselors undergo both generic and specialty training, para-counselors acquire basic general counseling and can offer basic help as they organize for a referral to the professional. They will participate in a 5-day training and receive monthly group supervision. Adaptation of G-CBT content, including cultural adaptation, will be ensured by intensive consultative meetings with the aforementioned stakeholders. During the consultative meetings, stakeholders will be introduced to the study and the core components of G-CBT for stigma, review manual/session content developed by the research team (based on a review of existing literature and CBT manuals utilized in Uganda and other settings), provide feedback on session content and cultural relevance, and make final revisions to the manual. The research team will ensure: (1) content adaptation of the materials to address stigma associated with mental illness to the context of HIV/AIDS-associated stigma, (2) developmental and cognitive adaptation of the content for children between 10 and 14 years, and (3) cultural adaptation of the G-CBT to local context. Content will be tailored to match children’s ability to comprehend and implement the therapeutic techniques, including age-appropriate activities, child friendly materials, simplified language and visuals (e.g., cartoons) [53], less complex behavioral techniques, and more support, structure and feedback [46]. In addition, the cultural adaptation is intended to enhance its treatment relevance, credibility, efficacy, and effectiveness by aligning it to the socioeconomic situation, cultural beliefs, family, political, and health systems in the region [54, 55].

For MFG, parent peers (*n* = 6) and community health workers (*n* = 6) already trained in MFG delivery focused on child behavioral health [42, 43, 50] will be invited to participate and receive a refresher training focused on Suubi4Stigma’s new content. Sessions focused on HIV and stigma will be adapted from curriculum used in our previous HIV-focused studies and resources from the Ministry of Health [41, 53]. The protocols have been designed to provide opportunities during each session to directly apply content to the realities of family life, emergent cultural and values perspectives, as well as tailor messages to age of child [43, 56]. These will include group activities, role plays, sharing experiences, and family take home activities. During MFG implementation, facilitators will receive 2 h per month of group supervision across sites.

### Implementation process evaluation

Process measures will be used to monitor fidelity and intervention implementation. For intervention delivery fidelity, independent observations (by trained research staff) using fidelity checklists will be made with a random sample of 60% of intervention sessions for both G-CBT and MFG. These data will be used to assess: (1) the relationship between planned and actual implementation; (2) integrity of implementation; and (3) how sessions were altered to maximize effectiveness and acceptability. In addition, having two facilitators per group will allow for detailed process notes on the implementation process and balancing “fidelity” and “fit” to local culture. Group participants will report monthly on factors that affect their participation using an implementation checklist that assesses satisfaction and obstacles to program delivery: (1) factors interfering with child or family participation (e.g., time, other priorities, stigma); (2) concrete obstacles (e.g., weather, transportation); and (3) site and staffing obstacles (e.g., time constraints).

### Quantitative data collection

Data will be collected at baseline, 3 months, and 6 months post-intervention initiation from both children and caregivers (see Table 2 for assessment measures). Interviewer administered interviews will be conducted by a trained research assistant at each clinic (in a private location) and take approximately 60–90 min with breaks and will be administered in Luganda, the local language widely spoken in the study region. Assessments will be translated and back translated into the local language by a certified translator.

**Table 2:** Assessment table

Variable	Measurement
***Respondent: children***	
Demographics	Socio-demographic questionnaire
Moderators	Economic/household income, gender, rural/urban/semi-urban
Mental health functioning	Adapted Child Depression Inventory [57]
	Adapted Tennessee Self-Concept Scale (TSC-2) [58]
	Beck Hopelessness Scale [59]
Post-traumatic stress disorder	Child PTSD Reaction Index [60]
Family support	Family Environment Scale [61]
	Family Assessment Measure [62]
	Adapted Social Support Behaviors Scale (SS-B) [63]
Social support	Friendship Qualities Scale [64]
Stigma and shame	Shame Questionnaire [65]
	Stigma-by-association scale for children [66]
	HIV Stigma mechanism measure [67]
Loneliness and social isolation	UCLA Loneliness Scale Version 3 [68]
Sexual risk taking	Adapted Youth AIDS Prevention Project used in CHAMP [69]
	Questions adapted from Auslander et al. [70] and Slonim-Nevo et al. [71]
Medication adherence	Youth-self-reports [72], viral load, pill counting, pharmacy records
Intervention feedback	Semi structured interviews
***Respondent: caregivers***	
Stigma and shame	AIDS-related stigma scale [73]
	Adapted Stigma-by-association measure [66]
Family relations	Family Environment Scale [61]
	Family Assessment Measure [62]
Mental health functioning	Adapted Brief Symptoms Inventory [74]
	Parenting Stress Index [75]
Intervention feedback	Semi-structured interviews

### Qualitative data collection

Our qualitative methods are informed by an interpretive approach that examines the experiences and practices of participants and their subjective meaning making processes [76]. Two sets of qualitative interviews with participants (children and caregivers) in the two treatment arms (G-CBT and MFG) will be conducted. The first set will be conducted at baseline to explore participants’ experiences with HIV-related stigma and its perceived impact on their lives and family wellbeing. The second interview will be conducted upon intervention completion to obtain feedback on intervention acceptability and relevance. Specifically, these interviews will (1) explore participants’ intervention experiences and perceived impact of the intervention on change mechanisms and (2) identify multi-level facilitators and barriers to G-CBT and MFG intervention, implementation, and participation. Our interview guides will be informed by the HIV stigma framework and the acceptability and appropriateness concepts developed by Proctor et al. to explore perceptions of intervention acceptability and impact [76]. The interviews will provide rich data on processes and experiences with the program; processes behind key outcomes, mechanisms of change, and mediating variables; and potential individual, family, contextual, programmatic, and structural factors affecting their experiences. Dyads with the highest, medium, and lowest scores on the stigma measures [66, 67, 73] in two treatment arms (6 clinics) will be identified at following baseline. Two families within each of these groups in the 6 treatment clinics will be invited to participate in qualitative interviews (2 × 3 × 6 = 36 dyads). This sampling method will ensure that participants with varying experiences are represented. This will allow us to identify common patterns and variations in participants’ experiences. The sample size will be sufficient for theoretical saturation [77, 78], and will allow for identification of common patterns and/or variations across participant experiences. In addition, all facilitators will be interviewed to gain a deeper understanding of implementation patterns and processes, including their views on sustainability. Interviews will be conducted in English or Luganda. Questions will be translated/back translated by two team members fluent in both languages. Interviews will last about 1 h and will be audio taped.

### Quantitative measures and analyses

#### Feasibility

We will monitor recruitment rates and staff level of effort, number of screenings conducted, as well as proportion eligible and agreed to enroll. Enrollment of 70% or higher will be considered feasible [79]. We will record the number of rescheduled, cancelled and missed G-CBT and MFG sessions, as well as assessments to inform estimation of staffing needs and retention protocols for a subsequent trial. We will also monitor attrition at each data collection point.

#### Acceptability

We will adapt satisfaction surveys, e.g., the Client Satisfaction Questionnaire (CSQ-8), to assess acceptability [80]. Some of the items include: “How satisfied were you with the program?”, “How helpful was the program in addressing HIV-associated stigma?” and “How likely are you to recommend this program to other families with CLWH?” Given the modest sample size, quantitative analyses of intervention data will be largely descriptive and concentrate on tabulating and summarizing satisfaction outcomes.

#### Preliminary impact

We expect that: H1: Following the intervention, relative to the control arm, participants in both treatment arms (G-CBT and MFG) will have: H1a: lower mean count of children reporting HIV-associated stigma; H1b: lower mean levels of reported HIV-associated stigma and improved child psychosocial functioning; H1c: lower mean levels of reported child mental health challenges; and H1d: higher mean levels of treatment adherence. H2: Following intervention, relative to the G-CBT intervention arm, participants in the MFG intervention arm will have: H2a: lower mean count of children reporting HIV-associated stigma; H2b: lower mean levels of reported HIV-associated stigma and improved child psychosocial functioning; H2c: lower mean levels of reported child mental health challenges; and H2d: higher mean levels of treatment adherence. Children will be the unit of analysis for these primary preliminary analyses.

Linear mixed models (LMMs) will be used to evaluate the proposed hypotheses. In addition, we will plot means by group over time to describe overall patterns of change. We will fit three-level LMMs including random intercepts for clinic membership and random intercepts and slopes for subjects. Due to the modest sample size, significance testing will be de-emphasized. Similarly, although the modest sample size precludes formal investigation of moderation, we will apply the same LMM approach described above to compare children across study arms over time on the moderators listed in Table 2. These moderation analyses will be secondary exploratory analyses. Additional exploratory analyses will study caregivers and children jointly as the unit of analysis via dyadic analysis methods such as actor-partner and means-and-deviation models to quantify caregiver vs. child stigma effects and between- vs. within-dyad effects on mental health outcomes [81, 82].

### Sample size and power calculation

Although the study purpose is to determine feasibility and acceptability, rather than conduct formal hypothesis tests, we conducted several power analyses using NCSS PASS to supply additional information. Assuming α = .05 and power = .80, 81 participants would be retained at the final time point following 10% estimated attrition, and a clinic-based conservative unconditional ICC of 9.3% based on our previous Suubi study implemented among children orphaned by HIV/AIDS in the study region [83]. Therefore, our sample size of 90 dyads provides adequate statistical power to test our primary aim of feasibility. Additionally, for the target enrollment proportion of 70% to assess feasibility, the width to the limit of the confidence interval is 27.9% (standardized distance .32). For continuous standard normal variables to assess acceptability, the distance from the mean to the confidence limit is .30. These distances to confidence limits are between small (.20) and medium (.50) effect sizes. For preliminary efficacy exploratory analyses with two time points and paired comparisons of two out of the three groups at 81/3 = 27 participants per group (*N* = 54 per comparison), minimum detectable standardized mean differences for continuous outcomes (stigma, mental health, and social support measures as described in Table 2) ranged from .79 to .97 for within-subject correlations (*r*) ranging from .20 to .80. Overall, our study is powered to detect small-medium distances to confidence limits for descriptive statistics and large longitudinal analysis effects.

### Qualitative data analysis

All interviews will be audio-taped, transcribed, and uploaded to QSR NVivo12 analytic software. Transcripts will be reviewed by the research team to develop a broad understanding of the content and identify topics of discussion and observation. Analytic induction techniques [84] will be used for coding. For initial coding, 10 transcripts will be randomly selected and read multiple times and independently coded by the team using a priori (from the interview guide) or emergent themes (open coding) [85]. Broader themes will be broken down into smaller, more specific units until no further subcategory is necessary. Analytic memos will be written to further develop categories, themes, and subthemes, and to integrate the ideas that emerge from the data [85, 86]. The codes and the inclusion/exclusion criteria for assigning a specific code [87] will be discussed as a team to create the final codebook in NVivo. Each transcript will then be coded independently by two team members using the codebook to establish inter-coder reliability. A level of agreement between 66 and 97% indicates good reliability [88]. Disagreements will be resolved through discussions in team meetings. The secondary analysis will compare themes within (including children versus caregivers) and across the two treatment groups to identify patterns, differences, and relationships among findings. Facilitators’ data will be analyzed using the same procedures and will be compared and contrasted to participant data. To further ensure rigor, member checking to explore the opinions, beliefs and attitudes of participants, data audit trail, and analytic memos will be used [78, 89].

### Data integration

Although qualitative and quantitative data analyses will be done separately, findings will be integrated at the interpretation and discussion stages. Conclusions and inferences will be synthesized for a more contextualized and thorough understanding of change mechanisms and the preliminary impact of each intervention arm. Specifically, qualitative and quantitative data will serve two purposes: (1) complementarity and (2) expansion [90, 91]. Qualitative findings will be connected to quantitative findings where the former will provide explanations and context for findings produced by the latter. Moreover, qualitative findings will complement our understanding of attendance and participant satisfaction for each treatment arm.

### Data safety and monitoring

A Data and Safety Monitoring Board (DSMB) will not be needed for this study because of the following: (1) *Suubi4Stigma* is a pilot intervention study with children and their caregivers in a single site; (2) We are using two evidence-based interventions, with no known adverse effect on their recipients; and (3) the interventions are psychosocial in nature and poses no more than minimal risk to participants. We believe that the multiple principal investigators (MPIs) (Nabunya and Ssewamala), In-country-PI (Mugisha), and Institutional Review Boards (IRBs)—both in Uganda and at Washington University in St. Louis, will be sufficient to monitor the trial. The MPIs have put in place a detailed Data and Safety Monitoring Plan (DSMP, details below) to ensure data safety.

To protect the integrity of participants’ data, the following procedures will be followed: first, all data collected from the study participants will be used only for the purpose of research. All data will be kept confidential. We will not share any information or answers we get from participants with their families, classmates, friends, or teachers. In the same way, we will not share any information or answers we get from the primary caregivers with their children, other relatives, friends, teachers, or public officials. Second, all participants (adolescents and caregivers) in the study will be assigned a random code number by the in-country study coordinator under the guidance of the MPIs. This code number is used on all information collected from participants, including questionnaires. We will maintain lists of participants with links between identifying information and code numbers to facilitate participant follow-up. Only the MPIs and in-country study coordinator will have access to these lists, which are kept in locked files. Other study personnel have access on an as needed basis to individual participants’ names and code numbers in order to adequately perform their duties, for example, interviewers must label the questionnaires with the correct code number of the participant whom they are interviewing.

All study personnel must complete certain levels of training before they are granted access to this identifying information. They must complete the Human Subjects Training. Personnel also sign confidentiality statements that specify that if the participants’ confidentiality is breached unintentionally that personnel will follow the procedures for reporting this breach to the MPIs. The confidentiality statements also state that unintentional or deliberate violations of participants’ confidentiality may result in demotion or termination depending upon the severity of the event. The project personnel also participate in training with the MPIs, the in-country study coordinator and the in-country PI regarding data safety, confidentiality of participants, limits of confidentiality, and proper administration of the study protocol.

All hard copies of data will be stored in locked cabinets to which only the MPIs and the in-country study coordinator will have access. After completion of an interview with a study participant, data with code numbers is placed in a separate locked file cabinet while waiting for entry. Once data is entered into computer files and password protected, only the MPIs, the in-country study coordinator and data entry assistants will have access to these files. All requests, current and future, to use the data are reviewed by the MPIs. Any data files that are provided to other individuals are stripped of identifiers and contain only code numbers so that data across multiple assessment waves can be matched.

Within the informed consent/assent, participants are notified of the above procedures. Participants are also informed of the limits of confidentiality. Specifically, participants are warned that Ugandan law mandates reporting of abuse and/or neglect of children, and that threat of harm to self or others requires intervention by clinical staff. Participants will be informed of these limits during the consent process and at the beginning of the interview process. To make sure that interviewers have accurate knowledge of what does and does not constitute reportable child abuse and/or neglect, interviewers will receive training on the Ugandan laws regarding child abuse and/or neglect. Interviewers who suspect child abuse and/or neglect will be instructed to contact the in-country study coordinator, the in-country PI, and/or MPIs, rather than contact the Ugandan local authorities themselves. Prior to making a decision of whether to make a report of child abuse and/or neglect, the case will be discussed among a group consisting of the interviewer, study coordinator, and the MPIs. It is important to note that all three MPIs are social workers by training and are knowledgeable about what types of child abuse and/or neglect rise to the level required for a report to be made. These procedures are intended to protect the safety of children, and at the same time reduce the risk that erroneous reports are made. If we determine that a report must be made, we will inform the caregiver of our intention to report, and the reasons why a report must be made, unless we think that doing so would pose an immediate risk to the youth. In case further counseling is needed, the project staff will make the referrals.

### Monitoring and responding to adverse events

All study personnel based in Uganda will be trained in identifying indicators of conditions that may jeopardize the welfare of participants and the limits of confidentiality. This training, conducted by the MPIs, includes reviewing possible scenarios and knowledge of key questions used to assess risk. Interview staff are trained to err on the side of caution and told to contact the study coordinator, who will always be available, by telephone, in the event of the need to break confidentiality due to mandatory reporting or ethical concerns. Under the guidance of in-country PI, research staff are trained either to contact the police to ensure safety of participants, or if appropriate, to have emergency personnel take the youth or caregiver to the nearest hospital.

Reporting of adverse events will occur according to a project protocol. For this study, safety and monitoring will be overseen by the in-country PI (Dr. Mugisha), and the in-country Co-I (Dr. Mwebembezi)—both of whom will be stationed in Uganda during the study, and the other two MPIs (Drs. Nabunya and Ssewamala, based in the USA). In the case of an adverse event, staff will inform the in-country PI (Dr. Mugisha) and Co-I, Dr. Mwebembezi immediately and then the MPIs Drs. Nabunya and Ssewamala within 24 h of the presence of a possible unanticipated adverse event. Any presence of a possible unanticipated adverse event will be immediately reported and brought to the attention of the Washington University Institutional Review Board (along with the Ethics Committee at Uganda Virus Research Institute, and Uganda National Council of Science and Technology). The IRBs will determine whether it is appropriate to stop the study protocol temporarily or will provide suggestions and/or modifications to the study procedures. Possible modifications may include adding new risks to the consent form and re-consenting all study participants.

Preliminary outcomes data will be examined quarterly by the MPIs, in-country PI, and the co-investigators. If preliminary outcome data indicates harmful impact of the program to the study participants (the children and/or their caregivers), Washington University IRB committee, as well as the Ethic Committee at Uganda Virus Research Institute and Uganda National Council of Science and Technology IRB will be notified and it is possible that the study will be discontinued immediately. However, we do not anticipate any negative effects of

### Data management and quality assurance

To collect data, Qualtrics, an electronic data capture system will be used. All tablets/iPads containing Qualtrics will be password protected. However, the option of completing a paper interview will remain available in case of technical difficulties. The interviewer will submit the tablet/iPad to their supervisor who will store the tablet/iPad in a locked file box until return from the field. Responses from Qualtrics will be downloaded daily by the data manager, onto a state-of-the-art secured dedicated server. All completed surveys will be printed out upon interviewers’ return from the field. These hard copies will be stored as back up of the data in a double-locked environment. Trained, supervised research assistants will enter the data onto secure, encrypted computers. The entered data will then be double-checked for completeness and accuracy. To address concerns of breach of confidentiality, all study documents and data will be maintained in password-protected computer files. Confidentiality of study documents will be maintained by assigning unique study IDs and using these rather than participant names on all study related materials. Paper copies of documents will be maintained in locked file cabinets in respective countries. Participant consent forms and ID logs will be kept in a separate locked cabinet. Tracking information also will be kept on a password-protected computer. The master list will only be used to coordinate data collection, and all staff will be required to receive training on both human subjects’ protections, as well as maintaining the confidentiality of participants. The MPIs and the in-country study coordinator will oversee the quality assurance monitoring.

#### Missing data

Research assistants and study coordinator will be trained to check assessments for missing items before leaving the participant. If any missing items are identified, participants will be asked to answer those items–unless they chose not to answer those particular items. The study coordinator will also review assessments within 5 working days of completion. If missing items are identified in the baseline assessment (time point 1), research assistants will follow-up with the participants before the first session of the intervention. If missing items are identified at follow-up time points (3 and 6 months post-intervention initiation), research assistants will follow-up with the participants within 7 days of the original date of the assessment.

#### Plan for record keeping

The study will maintain records of adverse events, any referrals for counseling, as well as copies of the consent and assent forms. All records will be maintained in a locked filing cabinet at the Uganda-project office accessible only by the research team. The MPIs will be responsible for data security and record keeping. The data sets that will be used for analysis will not contain any identifying information—specifically, names and addresses of the participants.

#### Plan for disposition of identifiers at the end of the study

Identifiers for the participants will be disposed of following local IRB guidelines. To protect the participants’ confidentiality, identifiers only will be accessible by the MPIs and Co-Is (Uganda- and US-based) and the study coordinator and will be kept separated from others with the participants’ responses.

## Discussion

This study seeks to address the urgent need for innovative, theoretically, and empirically informed interventions to reduce HIV/AIDS-associated stigma and its negative impact on adolescent health and psychosocial well-being. More specifically, the study will examine two evidence-based interventions used in mental health settings, schools, and communities: G-CBT focused on cognitive restructuring and strengthening coping skills at the individual level; and MFG intervention that strengthens family relationships to address stigma among CLWH and their families. Existing interventions focus on reducing fear of HIV infection among non-infected individuals as they interact with PLWH. This study offers an opportunity to develop a culturally acceptable and effective family-level intervention to address HIV/AIDS-associated stigma and its impact on CLWH’s wellbeing in SSA.

This study innovates in the following ways: (1) HIV stigma-reduction interventions targeting CLWH in SSA are almost non-existent. This study will generate data driven knowledge to address HIV-associated stigma among CLWH and within their families. (2) The study will apply and compare two evidence-based, theoretically guided interventions, G-CBT vs MFG, to address HIV-associated stigma. (3) The MFG approach is culturally consistent with SSA’s collective approach of families raising children “together,” which strengthens its appeal to communities and its likelihood of success in addressing both individual-and family-level stigma. (4) Delivery of G-CBT, which will be facilitated by trained para-counselors, is an approach that has not been tested in this context and with this specific population. In Uganda, para-counselors are trained to assist with the psychological needs of individuals, including those related to HIV/AIDS and mental health. (5) Partnering with local institutions, including health clinics and community organizations, grounds the project with a practical understanding of the needs of CLWH in Masaka, a region hardest hit by the HIV/AIDS epidermic.

Study findings will be disseminated through local, national, and global meetings/workshops/conferences and peer-reviewed publications. The research team will also hold meetings to share study results with community stakeholders in the study region. This study will lay the foundation for a larger intervention study investigating how HIV/AIDS-associated stigma can be reduced to foster healthy child development, especially for CLWH as they transition to adolescence. The long-term goal is to develop culturally appropriate, feasible, acceptable, and effective interventions that address HIV/AIDS-associated stigma and its impact on CLWH’s wellbeing and their families in SSA.

## Data Availability

N/A

## Abbreviations

ART: Antiretroviral therapy
CLWH: Children living with HIV
CSQ: Client Satisfaction Questionnaire
DSMB: Data and Safety Monitoring Board
DSMP: Data Safety and Monitoring Plan
G-CBT: Group Cognitive Behavioral Therapy
HIV: Human Immunodeficiency Viruses
ICHAD: International Center for Child Health and Development
IRB: Institutional Review Board
LMM: Linear mixed models
MFG: Multiple family groups
MPI: Multiple principal investigator
PI: Principal investigator
PLWH: People living with HIV
PTSD: Post-traumatic stress disorders
RA: Research assistant
RCT: Randomized controlled trial
RTY: Reach the Youth
SSA: Sub-Saharan Africa
